# Feasibility and Cost-Effectiveness of Treating Multidrug-Resistant Tuberculosis: A Cohort Study in the Philippines

**DOI:** 10.1371/journal.pmed.0030352

**Published:** 2006-09-12

**Authors:** Thelma E Tupasi, Rajesh Gupta, Ma Imelda D Quelapio, Ruth B Orillaza, Nona Rachel Mira, Nellie V Mangubat, Virgil Belen, Nida Arnisto, Lualhati Macalintal, Michael Arabit, Jaime Y Lagahid, Marcos Espinal, Katherine Floyd

**Affiliations:** 1 Tropical Disease Foundation, Manila, Philippines; 2 Stop TB Department, World Health Organization, Geneva, Switzerland; 3 Stanford University School of Medicine, Stanford, California, United States of America; 4 Infectious Disease Office, National Center for Disease Prevention and Control, Department of Health, Manila, Philippines; University of California San Francisco, United States of America

## Abstract

**Background:**

Multidrug-resistant tuberculosis (MDR-TB) is an important global health problem, and a control strategy known as DOTS-Plus has existed since 1999. However, evidence regarding the feasibility, effectiveness, cost, and cost-effectiveness of DOTS-Plus is still limited.

**Methodology/Principal Findings:**

We evaluated the feasibility, effectiveness, cost, and cost-effectiveness of a DOTS-Plus pilot project established at Makati Medical Center in Manila, the Philippines, in 1999. Patients with MDR-TB are treated with regimens, including first- and second-line drugs, tailored to their drug susceptibility pattern (i.e., individualised treatment). We considered the cohort enrolled between April 1999 and March 2002. During this three-year period, 118 patients were enrolled in the project; 117 were considered in the analysis. Seventy-one patients (61%) were cured, 12 (10%) failed treatment, 18 (15%) died, and 16 (14%) defaulted. The average cost per patient treated was US$3,355 from the perspective of the health system, of which US$1,557 was for drugs, and US$837 from the perspective of patients. The mean cost per disability-adjusted life year (DALY) gained by the DOTS-Plus project was US$242 (range US$85 to US$426).

**Conclusions:**

Treatment of patients with MDR-TB using the DOTS-Plus strategy and individualised drug regimens can be feasible, comparatively effective, and cost-effective in low- and middle-income countries.

## Abstract 2

Evaluation of 117 patients enrolled in a DOTS-Plus pilot project in the Philippines showed that in this setting the strategy is feasible.

## Editors' Summary

**Background.:**

Tuberculosis (TB) causes the death of some 2 million people each year. An estimated one in three people worldwide are infected with *Mycobacterium tuberculosis,* the bacterium that causes the disease. Because single-drug treatment leads to treatment failure and antibiotic resistance, treatment for active TB is complicated, usually involving four different antibiotics, at least two of which are continued for six months or more. The World Health Organization (WHO) recommends a specific strategy (DOTS) for diagnosing and treating TB (see Web link below).

The DOTS approach includes standard regimens of first-line drugs which cure about 90% of patients with drug-susceptible TB, and which cost as little as US$10 per patient. Unfortunately, TB resistance to at least two of the most effective DOTS drugs has developed at sites in both industrialized and developing countries, causing approximately 460,000 cases of multidrug-resistant TB (MDR-TB) per year. Second-line antibiotics, which tend to be more expensive or more difficult to take, can effectively treat many cases of MDR-TB. “DOTS-Plus” programmes, which use combinations of first- and second-line drugs to treat MDR-TB, are therefore becoming increasingly important in controlling TB worldwide. A recent study found DOTS-Plus strategies to be cost-effective in Peru, but cure rates of MDR-TB were relatively low.

**Why Was This Study Done?:**

Because the use of second-line antibiotics is costly and the treatment of MDR-TB has a higher failure rate than that of fully drug-susceptible TB, policymakers responsible for allocation of limited healthcare resources need information on how well DOTS-Plus programmes work and how much they cost to operate. This study was undertaken to assess the feasibility, effectiveness, and cost-effectiveness of a DOTS-Plus project in the Philippines, a lower middle–income country with a high rate of TB and approximately 25,000 cases of MDR-TB.

**What Did the Researchers Do and Find?:**

The researchers reported on a DOTS-Plus pilot project at Makati Medical Center in Manila, analyzing information from 118 patients enrolled in the project between 1999 and 2002. The diagnosis of MDR-TB was based on laboratory culture and antibiotic resistance testing of specimens from patients who had continued symptoms of TB following DOTS treatment, or other evidence of possible MDR-TB. Patients were treated with five-drug combinations individually selected based on resistance testing results, and administered under direct observation. After cultures had remained consistently negative for six months, patients were switched to a four-drug regimen with intermittent clinic observation until cultures remained negative for at least 18 months.

Cost-effectiveness was assessed by comparing the costs and effects of the project to the costs and effects that would have applied in the absence of the project, namely, no treatment of MDR-TB (except what patients could have purchased privately), or standard first-line DOTS treatment (which would not cure the majority of patients with MDR-TB, and is associated with a high chance of relapse in those who do appear cured). Costs of the DOTS-Plus project were based on expenditure records, project records, and interviews with staff, patients, and funding agencies. Effects of the project were based on treatment outcomes observed among enrolled patients, as well as on data on long-term outcomes among patients treated for MDR-TB in the US who were followed for up to ten years. Treatment costs for the situation in which no DOTS-Plus project exists were estimated using national data reported to WHO, as well as questionnaires administered to local patients in whom DOTS treatment had failed. Treatment outcomes where DOTS-Plus is not available were estimated from studies done in other TB-affected countries.

The researchers found that the cure rate of MDR-TB in this project was 61%. The cost per patient treated was US$4,192. They also calculated that the cost-effectiveness of the DOTS-Plus strategy was US$242 per disability-adjusted life year (DALY) gained, of which US$179 was paid by the healthcare system.

**What Do These Findings Mean?:**

The cure rate for MDR-TB in this project compares favourably to rates in other resource-limited settings where second-line TB drugs are used, and is much higher than in areas where these drugs are not available. From the standpoint of efficacy and patient well-being, then, this study supports the necessity of DOTS-Plus treatment. In purely economic terms, the cost of US$200–US$250 per DALY gained is cost-effective in comparison with other healthcare interventions. Specifically, because the gross national income per person in the Philippines is US$1,080, someone who can return to work following MDR-TB treatment costing US$250 per year gained of working life will provide work that is worth four times more, on average, than the cost of the treatment.

Although this study provides encouraging confirmation that DOTS-Plus programmes can be effective and cost-effective in a resource-limited setting, these findings are subject to several limitations. First, the data used to estimate treatment outcomes and the costs associated with chronic MDR-TB when DOTS-Plus treatment is not available were limited. Also, the pilot project in this study included only 118 of 171 eligible patients, leaving open the possibility that the other 53 patients might have had different outcomes. In addition, the long-term relapse rate in the treated patients is unknown. Finally, the conclusion that one model programme is effective does not mean that other programmes will do well under less favourable circumstances. Nonetheless, as MDR-TB continues to spread in the developing world, a good example is good news. A Perspective by Paul Garner and colleagues in this issue of *PLoS Medicine* (DOI: 10.1371/journal.pmed.0030350) discusses the study further.

**Additional Information.:**

Please access these Web sites via the online version of this summary at http://dx.doi.org/10.1371/journal.pmed.0030352.

- Basic information about tuberculosis can be found on the Web site of the US National Institute of Allergy and Infectious Diseases (NIAID)
- The Web site of the World Health Organization's Stop TB department outlines both the  DOTS and DOTS-Plus strategies
- TB Alert, a UK-based charity that promotes TB awareness worldwide, has information on TB in several European, African, and Asian languages

## Introduction

Multidrug-resistant tuberculosis (MDR-TB), which is defined as resistance to at least the two most effective first-line anti-TB drugs, rifampicin and isoniazid, is a threat to tuberculosis (TB) control efforts in some countries [1–3]. Approximately 460,000 cases develop each year, about half of them among new TB cases and the other half among people who have been previously treated for TB [4]. When only first-line anti-TB drugs are used, cure rates among patients with MDR-TB are low: 5%–35% for previously treated cases and 12%–60% for new cases [5], with a relapse rate of 24% [6].

Treatment that includes second-line anti-TB drugs can considerably improve cure rates [7–20] and reduce transmission, but its availability remains limited in low- and middle-income countries. This reflects the high price of second-line drugs [21] as well as the (related) emphasis of national and international TB control efforts on implementation of the DOTS strategy. The DOTS strategy focuses on treatment of the approximately nine million new cases of TB that occur each year with standardized first-line drug regimens, which cure about 90% of patients in the absence of MDR and cost as little as US$10 per patient [22].

Building on the successes of DOTS, the World Health Organization (WHO) and partner agencies developed a strategy for treatment of MDR-TB, termed “DOTS-Plus,” in 1999 [21,23]. The DOTS-Plus strategy adapted the core components of DOTS to the needs of patients with MDR-TB, in particular including diagnosis based on culture and drug susceptibility testing and treatment with second- as well as first-line drugs. As it was recognized that the high price of second-line drugs would be a major impediment to implementation of the DOTS-Plus strategy, simultaneous efforts were made to lower the price of second-line drugs. These efforts resulted in the Green Light Committee (GLC) [21,24], which in partnership with pharmaceutical companies is able to provide second-line drugs at highly concessionary prices to projects and programmes that meet strict eligibility criteria.

A growing number of low- and middle-income countries are now implementing DOTS-Plus pilot projects or programmes, with 33 GLC-approved projects by the end of 2005. However, evidence about their feasibility, effectiveness, cost, and cost-effectiveness remains scarce. An evaluation of the national programme in Peru showed that treatment of chronic cases, 87% of whom had MDR-TB, was feasible and cost-effective, though cure rates were relatively low [8]. Published data on treatment outcomes are limited to Latvia, Peru, and Turkey [8,9,11–13]. More data are required for policy development, planning, and budgeting at global and national levels.

In April 1999, a DOTS-Plus pilot project was initiated at the Makati Medical Center (MMC) in Manila, Philippines [25]. This article assesses the project's feasibility, effectiveness, cost, and cost-effectiveness.

## Methods

### Setting

Philippines is a lower middle−income country with a per capita gross national income of US$1,080 in 2003 [26]. Globally, it ranks eighth in terms of the estimated number of new TB cases that occur each year, with about 240,000 cases in 2004 [4]. It is estimated that there are 25,803 MDR-TB cases: 7,238 new cases and 18,565 previously treated cases [3,27].

DOTS is implemented nationally, with a case detection rate of 73% and a successful treatment rate of 89% for new smear-positive cases [4], both in excess of WHO targets. DOTS treatment involves a four-drug first-line regimen for new cases, and a five-drug retreatment regimen for patients who fail treatment with this first regimen or who have had TB before and have suffered a relapse. Patients who fail the retreatment regimen are defined as chronic cases [28], and as of March 2006, no treatment was available for them in the public sector. Treatment in the private sector is generally of unknown quality and limited by patients willingness and ability to pay.

MMC is a private tertiary hospital in the main commercial district of Manila. It established DOTS services in 1999, in partnership with the Department of Health and the local government [29]. A DOTS-Plus pilot project was started in April 1999. In March 2006, MMC remained the only facility in the country offering such treatment.

### Description of the DOTS-Plus Pilot Project

Two major categories of TB cases were eligible for treatment in the DOTS-Plus project: (a) chronic cases of MDR-TB referred from public or private facilities; and (b) cases with a diagnosis of MDR-TB during treatment with the first-line retreatment regimen. A few patients with MDR-TB identified among new cases during contact tracing or treatment with first-line drugs were also enrolled. Diagnosis was based on smear and culture examination. After informed written consent, patients were treated with an individualised regimen based on drug susceptibility testing results for all first-line drugs, three second-line drugs (kanamycin, ciprofloxacin, and ofloxacin), and previous use of other drugs as reported by patients. In the intensive phase of treatment, a daily five-drug regimen was used. This typically consisted of an injectable drug, a fluoroquinolone, other oral second-line drugs, and first-line drugs to which the patient was not resistant. In the continuation phase, started after six consecutive months of negative culture results, the injectable or (occasionally) a noninjectable to which the patient was intolerant was dropped from the regimen. Treatment was continued until cultures were negative for 18 consecutive months. During the intensive phase, direct observation of treatment (DOT) was provided by MMC staff. In the continuation phase, alternating clinic and home-based DOT was used. Patients who defaulted were followed up by telephone, telegram, and/or home visits.

### Patient Cohort Studied

We considered the patient cohort enrolled between 1 April 1999 and 31 March 2002.

### Treatment Outcomes

Treatment outcomes for the DOTS-Plus project were assessed using internationally agreed consensus definitions [30]. There were six possible outcomes: cured, completed treatment, died, defaulted, transferred out of the district and treatment outcome unknown, and failed treatment. Cure was defined as having completed at least 18 months of treatment, with negative cultures in the last month of treatment and consistent culture-negative status for the previous 11 months. Completed treatment meant that a patient was regarded to have been cured, but bacteriological tests to confirm this were not available. The outcome “died” was applied for both TB and non-TB related deaths. Failed meant that the patient remained culture-positive at the end of treatment.

### Cost and Cost-Effectiveness Analysis

Any cost-effectiveness analysis requires comparison of relevant alternative strategies [31]. We compared the DOTS-Plus project with the situation that would apply in the absence of the project, i.e., what would have happened to the cohort of DOTS-Plus patients had they not been enrolled in the project. For chronic MDR-TB cases, this meant no treatment, or limited treatment based on what patients could afford to pay for in the private sector. For retreatment cases with MDR-TB, it meant continued use of the standard first-line retreatment regimen. For new cases with MDR-TB, it meant treatment with the standard first-line drug regimen.

Costs were assessed in year 2002 US$ from a societal perspective (i.e., health system, patient, and family costs were considered) using standard methods [31,32].

For the DOTS-Plus project, two types of costs were considered: (a) the average cost of individual components of treatment (e.g., drugs, a DOT visit); and (b) the average cost per patient treated. The costs of individual components of treatment were calculated using an “ingredients” approach, i.e., the quantity of resources used was multiplied by unit prices. Joint costs (e.g., clinic staff that spent time on both DOTS and DOTS-Plus patients) were allocated according to the time spent on each group of patients. Vehicle and equipment costs were annualised using current replacement prices, the assumption of a five-year life expectancy, and a discount rate of 3% [33]. Startup training costs were annualised over three years. Building costs per year were based on rental values per month. All local costs were converted into US$ using the average exchange rate in 2002 (US$1 = PhP 50.9). The average cost per patient treated was calculated as the cost of each treatment component multiplied by the average number of times this cost was incurred. Sources of data included expenditure records, interviews with staff and patients, project records and databases, and the GLC secretariat.

To assess treatment costs for chronic cases in the absence of a DOTS-Plus project, all retreatment failures registered in three administrative units of Metro Manila during 2000 and 2001 were identified. The three areas were purposively selected based on good DOTS programme performance and a large population size. The 46 treatment failures that were identified were traced, and they or a close relative (if the patient had died) interviewed about any further treatment that had been taken and related expenditures, using a standardized structured questionnaire. Costs for new and retreatment cases were estimated using national data reported to WHO [4].

Given uncertainty about several parameters that influence effectiveness, which in turn affects both total costs and cost-effectiveness, we estimated these three indicators as part of a multivariate uncertainty analysis. For consistency and comparability, this analysis was based on the same principles and much of the data that were used in the evaluation of the MDR-TB treatment programme in Peru, full details of which are available elsewhere [8]. In brief, the analysis was designed to measure the effectiveness of each strategy in terms of cases cured, deaths averted, and DALYs gained, and to capture both (a) effects among the patient cohort treated and (b) the effect of treatment of this cohort on transmission, and hence the number of cases, deaths, and DALYs gained that occur in the future. The analysis used an Excel spreadsheet model in which treatment paths were defined for the two alternative strategies (i.e., DOTS-Plus available, and DOTS-Plus not available). For each strategy, treatment paths were defined for chronic, retreatment, and new MDR-TB cases separately. The number of patients following each treatment path, together with their associated costs and effects, was defined according to (a) the parameters, parameter distributions, and data sources listed in Protocol S1 [2,4,5,34–43], (b) the costs per patient reported in this article, and (c) the treatment outcomes associated with the DOTS-Plus project, also reported in this article. As shown in Protocol S1, treatment outcomes for chronic cases when DOTS-Plus is not available were estimated from long-term follow-up studies of chronic cases in Korea, Vietnam, and the Russian Federation, and natural history studies. These outcomes were also assumed to apply to patients who defaulted from DOTS-Plus treatment. Long-term outcomes for patients treated in a DOTS-Plus project, including those who failed treatment or who relapsed after cure, were based on a study of MDR-TB patients with ten-year follow-up in the United States. Treatment outcomes for MDR-TB cases on first-line regimens were based on a multi-country study. A Monte Carlo simulation involving 5,000 iterations was used to estimate means, and lower and upper bounds (fifth and ninety-fifth centiles) for the main outputs of interest, i.e., the total costs and total DALYs lost for each strategy, the total DALYs gained by DOTS-Plus, and the cost per DALY gained by DOTS-Plus.

### Statistical Analysis

In addition to the uncertainty analysis described above, we compared the clinical and demographic characteristics of the cohort enrolled in treatment with patients who were eligible but not enrolled in treatment, using chi-square tests for categorical outcome variables and *t*-tests for continuous outcome variables. We also used the chi-square test to compare the treatment outcomes of chronic cases with those of new and retreatment cases. Given the small number of new cases (*n* = 5), we combined new and retreatment cases in one category when making comparisons with chronic cases.

## Results

### Patient Enrollment and Characteristics

**Figure 1:**
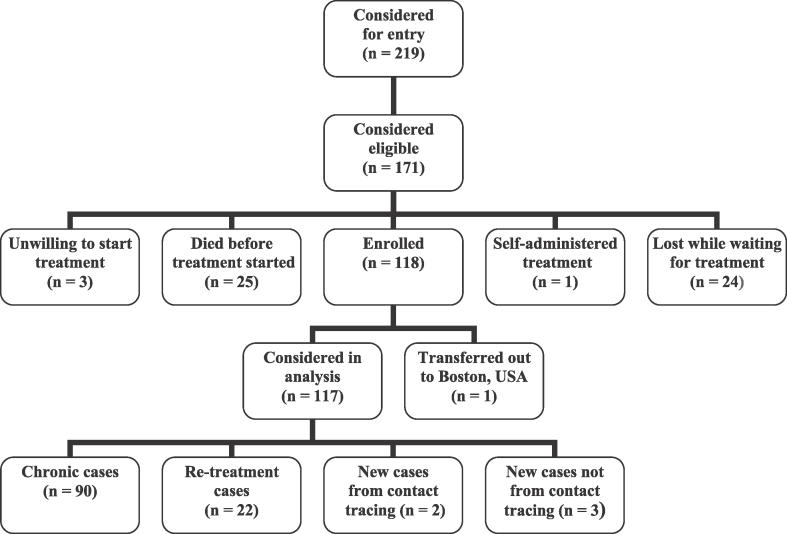
Patient Enrollment in DOTS-Plus Project, 1 April 1999 to 31 March 2002

Between April 1999 and March 2002, 219 cases were evaluated. Among these cases, 171 were confirmed to have MDR-TB. Of these 171 cases that were eligible for MDR-TB treatment, 118 were enrolled in the DOTS-Plus project, and 117 were considered in the analysis (Figure 1). A comparison of the 118 enrolled patients with the 53 considered eligible but who were not enrolled showed no significant difference in key characteristics such as age, sex, and number of previous treatments (Protocol S2). There was a significant difference in the percentage that were resistant to five or more drugs (*p* < 0.001), with more of those enrolled being resistant to five or more drugs, and in place of residence with more of those enrolled residing in Makati (*p* = 0.05).

The characteristics of the cohort are summarized in Table 1. Ninety were chronic cases, and 27 were new or retreatment cases. The average age was 38 years; 86 (74%) were male. The average income was US$31 per month (range US$0–US$625), and the average number of treatment courses before enrollment on DOTS-Plus was 2.8. Many patients (62%) were resistant to five or more drugs. Lack of testing services meant that the HIV status of patients was unknown, but 27 patients had diseases other than TB: diabetes (*n* = 17), chronic obstructive pulmonary disease (*n* = 3), peptic ulcer (*n* = 2), and heart disease (*n* = 2). Of the 102 patients with X-ray results, 66 had cavitary disease. Of the 117 patients, 106 had pulmonary TB and 11 had extrapulmonary TB. The characteristics of chronic cases on the one hand, and new and retreatment cases on the other, were generally similar. The main statistically significant differences (*p* ≤ 0.05) were that a much higher proportion of chronic cases had previously been treated with second-line drugs (*p* < 0.001), while a relatively high proportion of new and retreatment cases lived locally, in Makati (*p* < 0.001).

**Table 1:**
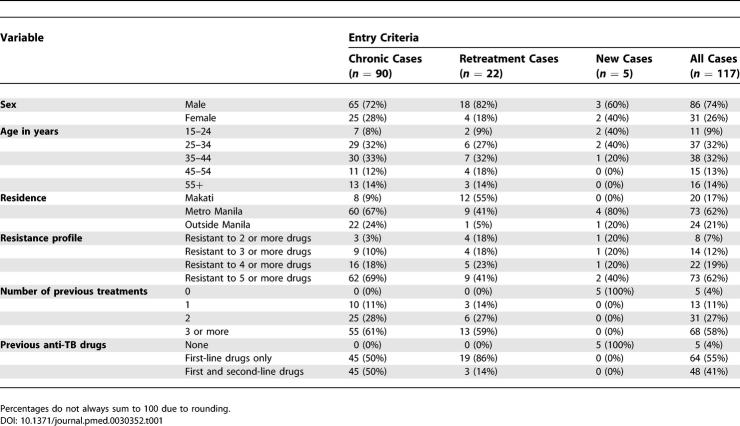
Demographic Characteristics and Disease-Related Variables according to Enrollment Criteria

### Treatment Outcomes and Adverse Events

Treatment outcomes are shown in Table 2. For the entire cohort, the cure rate was 61% (71/117, including 70 patients for whom cure was confirmed by bacteriological examination and one patient who was considered to have been cured but for whom bacteriological tests to confirm this were not available). Of the 71 patients cured, 90% had converted to sputum culture−negative status after three months of treatment. Of the 18 patients who died, nine of 14 (64%) investigated were bacteriologically negative at the time of death. Among the 16 patients who defaulted, eight of 15 (53%) investigated were bacteriologically negative when they defaulted. Default rates were lower among chronic cases compared with new and retreatment cases (*p* = 0.01).

**Table 2:**
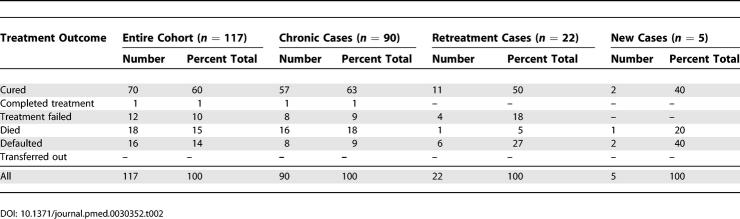
Treatment Outcomes for Patients Enrolled in DOTS-Plus, 1 April 1999 to 31 March 2002

Adverse events were the main reason for default (11/16). Two patients cited financial difficulties; the remaining three causes were family problems, a decision to return to a home located in another province, and a hospital discharge that was made without the endorsement of MMC staff. We could not identify any clinical variable that was a positive predictor of cure, though women appeared more likely to be cured than men.

During treatment, almost all (112/117, or 96%) patients experienced adverse events (Table 3). Most side-effects were minor, but serious side-effects such as hearing loss and depression were also observed. Side-effects were managed through treatment with ancillary drugs, through temporary interruption of the drug suspected to be the cause of the adverse event, or (among 49% of patients) through removing the suspected drug from the treatment regimen and replacing it with a suitable alternative. The name, class, and frequency with which drugs were used to treat patients, and their relationship with adverse events, is shown in Table 4.

**Table 3:**
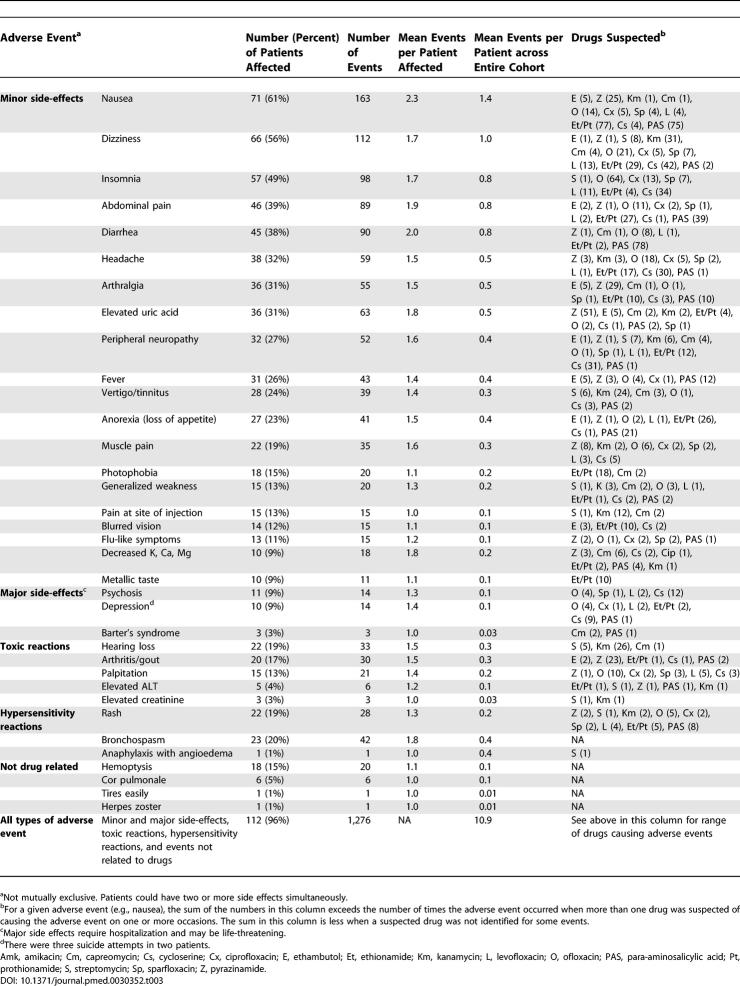
Adverse Events in 117 Patients Enrolled in DOTS-Plus

**Table 4:**
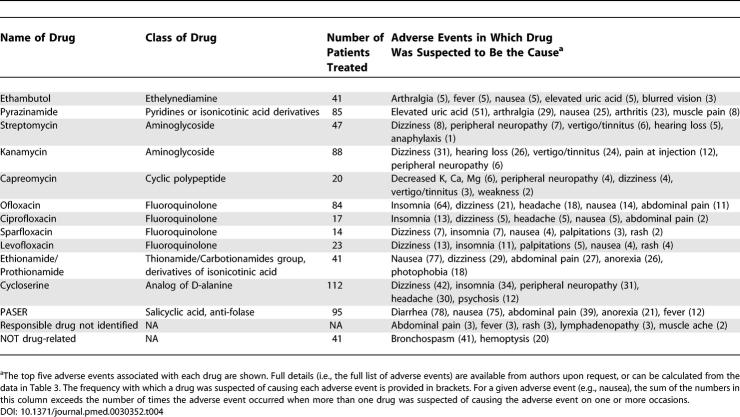
Drugs Used for DOTS-Plus Cohort, and Main Adverse Events with Which They Were Associated

### Cost and Cost-Effectiveness

The average cost per patient treated in the DOTS-Plus project was US$4,192, of which US$3,355 was health system costs and US$837 costs incurred by patients and their families (Table 5). Drugs, at US$1,557 per patient, were the most important cost item. At market rather than GLC prices, the average cost of drugs per patient would have been US$1,343 higher. For patients and their families, costs were mostly for clinic visits for DOT, and board and lodging. In the absence of a DOTS-Plus project, the average cost per MDR case was US$116. The average cost per chronic case was US$100, all out-of-pocket expenditures by patients. It was US$317 for retreatment cases and US$235 for new cases, with both costs roughly split between patients and the health system. Out-of-pocket expenditures by chronic cases in the private sector were mostly for prescriptions restricted to first-line drugs. Only six of the 46 chronic cases that were identified had prescriptions for second-line drugs, usually a fluoroquinolone.

**Table 5:**
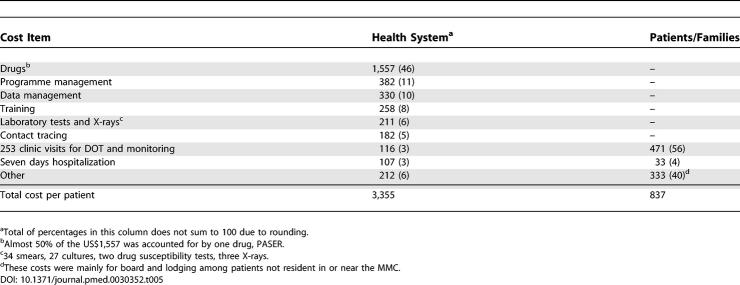
Average Cost per Patient Treated in 2002 US$ (Percent Column Total), DOTS-Plus Project

The total costs for each strategy, including both the costs of the cohort of 117 patients and costs associated with secondary cases generated through transmission of TB by this cohort, are shown in Table 6. The net increase in total costs associated with the DOTS-Plus strategy was about US$0.4 million, additional costs that resulted in a large number of averted deaths and DALYs gained (Table 6). The mean cost per DALY gained by DOTS-Plus from the health system's perspective was US$179, and US$242 when all costs were considered.

**Table 6:**
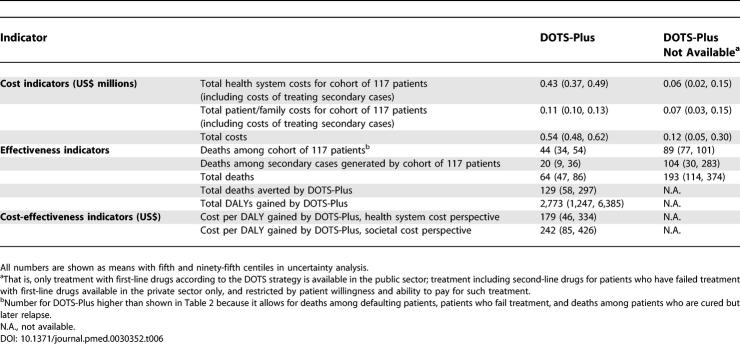
Cost, Effectiveness, and Cost-Effectiveness Indicators, Alternative Strategies

## Discussion

This study demonstrates that resource-limited settings can provide individualised treatment including second-line drugs at an appropriate standard of care for highly resistant MDR-TB patients: 86% of patients complied with treatment, and the cure rate was 61%, much higher than has been documented for chronic cases in low- and middle-income countries when DOTS-Plus is not implemented and treatment with second-line drugs is limited or not available [34–36]. At US$200–US$250 per DALY gained, DOTS-Plus in this setting is cost-effective when compared with benchmarks that are widely used to assess intervention cost-effectiveness, such as a cost per DALY gained of less than per capita gross national income (US$1,080 in the Philippines) or one to three times gross domestic product per capita [44,45].

The cure rate was towards the upper end of the 40%–75% range observed in other resource-limited settings where second-line drugs are used [8,9,11,13]. Considering the extensive drug resistance (62% of patients were resistant to ≥5 drugs), the frequency of adverse events, and the fact that most patients were chronic cases and had already received three or more previous treatments, the cure rate can be considered to be relatively good. In fact, it could be argued that given the frequency and nature of some adverse events (e.g., hearing loss, which is usually irreversible), the default rate of 14% could easily have been higher. The fact that counselling and education about adverse events was provided by MMC staff and that many adverse effects were categorized as “minor” may explain why most patients experiencing adverse events did not default. For chronic cases, a further important reason is that DOTS-Plus treatment is regarded as the last chance of being cured of TB; this may also explain why the default rate was lower than for new and retreatment cases, a finding that warrants further investigation.

Four measures have now been implemented to reduce the default rate: provision of ancillary drugs for management of adverse events free of charge to patients (previously, patients needed to pay for these drugs); decentralization of treatment so that it is available closer to where patients live, thus reducing costs; provision of psychosocial support; and use of patient incentives and enablers. Preliminary data suggest that these measures will lower the default rate to about 7%. This would also improve cost-effectiveness to about US$150 per DALY gained from a health system perspective and to about US$200 from a societal perspective.

The cost to the health system, at US$3,355 per patient, was more than the US$2,381 reported for the national DOTS-Plus programme in Peru, mostly due to the higher cost of drugs (US$1,557 versus US$824 in Peru) [8]. However, since higher costs were accompanied by higher cure rates in the Philippines (61% versus 48%), cost-effectiveness was similar, at about US$200 per DALY gained.

The study has several limitations. The project identified a large number of eligible patients who were not enrolled (53 out of 171). However, ten of these patients were enrolled after April 2002 and our comparison with the study cohort showed no significant differences in demographic or clinical characteristics besides residence and the percentage of patients who were resistant to five or more drugs (with more severe drug resistance among those enrolled). A major reason for non-enrollment—lack of funding at the time the patients were identified—was clearly recognized by MMC staff. The relapse rate is not yet known, though long-term follow-up is occurring. The applicability of the data to people with HIV infection and children is unknown, since the patient cohort was restricted to adults and rates of HIV infection were believed to be low. As in previous studies, data about the outcomes and costs associated with chronic cases of MDR-TB when DOTS-Plus treatment is not available were scarce. Nonetheless, considerable efforts were made to collect relevant data locally, in addition to making use of long-term follow-up studies from other countries. While recognizing the shortcomings of these data, we believe that they are the best that could be obtained without a long-term study. Furthermore, the costs reported in the absence of DOTS-Plus were very low; if anything we may have overestimated the increase in costs that occurs when DOTS-Plus treatment is made available.

Strengths of the study include the fact that it is only the second to our knowledge to provide evidence of the feasibility, effectiveness, cost, and cost-effectiveness of DOTS-Plus in a low- or middle-income country, and the first to report empirical data on the costs of treatment when individualised regimens are used. To our knowledge, it is also the first study to document costs from a patient and household perspective as well as a health systems perspective, the first from a GLC-monitored DOTS-Plus pilot project, and the first from Asia.

Nationwide expansion of DOTS-Plus in the Philippines is likely to start in 2006 or early 2007, following a successful round five application to the Global Fund to fight AIDS, TB, and Malaria (GFATM) that was approved in December 2005. Achieving treatment outcomes similar to those at MMC at other sites will require four elements of the MMC project to be replicated: a high level of commitment from all partners; technical support from international institutions such as WHO and the GLC; a clinic team that is highly motivated and committed to providing DOT, managing adverse events, and following up on defaulters; and a similar level of resources (i.e., about US$3,500 per patient enrolled in treatment). In parallel it is important to improve treatment for drug-susceptible cases in the private sector (the source of more than 75% of cases in the cohort that we studied), to prevent the problem of MDR emerging in the first place. Encouragingly, nationwide expansion of the public−private mix DOTS (PPMD) strategy is well under way [4].

Overall, our study demonstrates that treatment of patients with MDR-TB using the DOTS-Plus strategy and individualised drug regimens can be feasible, comparatively effective, and cost-effective in low- and middle-income countries. Success with nationwide scaling up in the Philippines will now depend primarily on the application of lessons learned during this [and the overall] DOTS-Plus experience.

## Supporting Information

Protocol S1 Provides Details of the Variables, Parameters, and Parameter Distributions Used for the Effectiveness and Cost-Effectiveness Analysis (68 KB DOC)

Protocol S2 Provides Data Comparing the Patients Who Were Enrolled in Treatment with the 53 Patients Who Were Eligible for Treatment but Who Were Not Enrolled (49 KB DOC)

## Abbreviations

DALY: disability-adjusted life year
GLC: Green Light Committee
MDR-TB: multidrug-resistant tuberculosis
MMC: Makati Medical Center
TB: tuberculosis, WHO, World Health Organization
