# Supplementary material for: Feasibility and Cost-Effectiveness of Treating Multidrug-Resistant Tuberculosis: A Cohort Study in the Philippines
Source: PLoS Med. 2006 Sep 12;3(9):e352. doi: 10.1371/journal.pmed.0030352 (PMC1564168; doi:10.1371/journal.pmed.0030352)
Supplement: Supplementary file 1 [file pmed.0030352.sd001.doc]

Protocol S1: Variables and variable values used to estimate effectiveness and cost-effectiveness

| Variable | Variable value (distribution where applicable)* | | | Source of data |
| --- | --- | --- | --- | --- |
| General variables related to effectiveness | | | | |
| Self-cure rate among chronic MDR tuberculosis patients (%) | 1 | | 34-36 | |
| Treatment outcomes for MDR tuberculosis cases on first-line re-treatment regimen  Cure rate (%)  Death rate (%)  Default rate (%)  Failure rate (%) | 35·0, 4·0 (normal)  12·9, 2·8 (normal)  11·4, 2·7 (normal)  1- (cure + death + default rate) | | 5 | |
| Treatment outcomes for new MDR tuberculosis cases treated with standard first-line treatment regimen  Cure rate (%)  Death rate (%)  Default rate (%)  Failure rate (%) | 57·9, 3·9 (normal)  9·8, 2·3 (normal)  8·5, 2·2 (normal)  1- (cure + death + default rate) | | 5 | |
| Long-term death rate for chronic MDR tuberculosis cases not receiving treatment with second-line drugs (%) | 60, 99 (uniform) | | 34-36,42 | |
| Long-term death rate from tuberculosis among MDR cases who fail treatment with second-line drugs (%) | 32, 6·8 (normal) | | 37 | |
| Long-term relapse rate among MDR tuberculosis cases initially cured with regimens that include second-line drugs (%) | 14, 3·7 (normal) | | 37 | |
| Death rate from MDR tuberculosis among cases initially cured with regimens that include second-line drugs, but who later relapse (%) | 100 | | 37 | |
| Death rate among patients who default (%) | Set to equal death rate for chronic MDR cases with no access to second-line drugs | | Assumption† | |
| Average survival of MDR tuberculosis patients who fail treatment with first-line drugs, in absence of treatment with second-line drugs (years) | 5·5 | | 34, 35, 42 | |
| Discount rate for valuing health gains arising in the future (%) | 3 | | 33 | |
| Average health gain associated with one averted death (DALYs)‡ | 21·5 | | 38,39 | |
| Variables specifically related to effects associated with the impact of treatment on transmission | | | | |
| Case reproduction number for MDR tuberculosis when no treatment with second-line drugs is available§ | | 0·5, 1 (uniform) | 2,43 | |
| Case reproduction number for MDR tuberculosis, in the presence of treatment with second-line drugs | | {(Average duration of infectiousness in presence of treatment ÷ average duration of infectiousness in absence of treatment) × Case reproduction number for chronic tuberculosis when no treatment with second-line drugs is available} | Assumption that amount of transmission reflects duration of infectiousness | |
| Generation time (years) | | 2, 4 (uniform) | 40 | |
| Total number of secondary MDR tuberculosis cases arising g years in the future, where g = generation time in years and R is the case reproduction number | | Re^-0.03×g^ | 40 | |
| Total number of secondary MDR tuberculosis cases arising in the future, where r is the total number of secondary cases arising g years in the future and g is the generation time in years i.e. r = Re^-0.03×g^ | | r ÷ (1-r) | Standard formula when r < 1 | |
| Average duration of infectiousness, chronic MDR tuberculosis case not treated with second-line drugs (years) | | 2 | 41 | |
| Average duration of infectiousness, chronic MDR tuberculosis case treated with second-line drugs whose outcome is death or default (years) | | 2, plus the period on DOTS-Plus treatment | Assumption¶ | |
| Average duration of infectiousness, chronic MDR tuberculosis case, when final outcome is treatment failure (years) | | 2, plus the period on DOTS-Plus treatment | Assumption¶ | |
| Average duration of infectiousness, MDR tuberculosis cases cured on first-line treatment or re-treatment regimen (years) | | 0·25 | Assumption | |
| Average duration of infectiousness, MDR tuberculosis cases who are cured on DOTS-plus (years) | | 0·23 | This study | |
| Death rate for secondary MDR tuberculosis cases on first-line treatment regimen among patients who default from this regimen (%) | | 7, 2·0 (normal) ) × proportion of untreated chronic cases that die | 5 | |
| Average health system cost per patient treated on first-line treatment regimen (US$) | | 174 | 4 | |
| Average cost to patients on first-line treatment regiment (US$) | | 119 | This study | |
| Average health system cost per patient treated on the re-treatment regimen (US$) | | 198 | 4 | |
| Average cost to patients on first-line treatment regimen (US$) | | 177 | This study | |

*where a uniform distribution is specified, this indicates that the analysis used values chosen at random between the lower and upper figures specified for the distribution. Where a normal distribution is specified, the first number specifies the mean and the second number the standard deviation that define the distribution. See text for further details.

†this assumption is justified on the basis that patients who default are no longer receiving treatment, and can therefore be expected to have treatment outcomes similar to those of chronic tuberculosis patients with no access to second-line drugs.

‡the formula for calculating DALYs is provided in the reference given (reference 38). Use of the formula requires data on the average age at onset of disease (in this case 30 years), and the average life expectancy at that age (in this case 40 years).

§the case reproduction number in the absence of treatment with second-line drugs was assumed to lie in the range 0.5 to 1 to be conservative when assessing cost-effectiveness. A less conservative assumption, which would improve (i.e. lower) cost per DALY gained figures, would have been to assume that the case reproduction number in the absence of treatment with second-line drugs would be equal to 1.

¶value is conservative with respect to effectiveness and cost-effectiveness calculations (i.e. value may lead to effectiveness being under-estimated) because the period on treatment is added to the duration of infectiousness expected in the absence of treatment.
