# Supplementary material for: Feasibility and Cost-Effectiveness of Treating Multidrug-Resistant Tuberculosis: A Cohort Study in the Philippines
Source: PLoS Med. 2006 Sep 12;3(9):e352. doi: 10.1371/journal.pmed.0030352 (PMC1564168; doi:10.1371/journal.pmed.0030352)
Supplement: Supplementary file 2 [file pmed.0030352.sd002.doc]

**Protocol S2: Comparison of the 118 patients enrolled on treatment with the 53 patients who were eligible but not enrolled**

| **Variable** | **Patients enrolled (n=118)** | **Patients eligible but not enrolled (n=53)*** | **p-value** |
| --- | --- | --- | --- |
| **Age** | | | |
| 15-24 | 11 | 4 | 0.30 |
| 25-34 | 38 | 12 |  |
| 35-44 | 38 | 15 |  |
| 45-54 | 15 | 13 |  |
| 55+ | 16 | 9 |  |
| **Sex** | | | |
| Male | 86 | 35 | 0.36 |
| Female | 32 | 18 |  |
| **Residence** | | | |
| Makati | 21 | 2 | 0.05 |
| Metro Manila | 73 | 34 |  |
| Outside Manila | 24 | 14 |  |
| **Mean number of previous treatments [95% CI]** | 2.82 [2.6,3.04] | 2.98 [2.49,3.47] | 0.30 |
| **Resistance profile** | | | |
| Resistant to less than 5 drugs | 45 | 41 | <0.001 |
| Resistant to 5 or more drugs | 73 | 12 |  |

*column sub-total does not add to 53 when data were unavailable for some patients.
